# Revisiting the embryogenesis of lip and palate development

**DOI:** 10.1111/odi.14174

**Published:** 2022-03-05

**Authors:** Nigel L. Hammond, Michael J. Dixon

**Affiliations:** ^1^ Faculty of Biology, Medicine and Health University of Manchester Manchester UK

**Keywords:** cleft lip, cleft palate, facial development

## Abstract

Clefts of the lip and palate (CLP), the major causes of congenital facial malformation globally, result from failure of fusion of the facial processes during embryogenesis. With a prevalence of 1 in 500–2500 live births, CLP causes major morbidity throughout life as a result of problems with facial appearance, feeding, speaking, obstructive apnoea, hearing and social adjustment and requires complex, multi‐disciplinary care at considerable cost to healthcare systems worldwide. Long‐term outcomes for affected individuals include increased mortality compared with their unaffected siblings. The frequent occurrence and major healthcare burden imposed by CLP highlight the importance of dissecting the molecular mechanisms driving facial development. Identification of the genetic mutations underlying syndromic forms of CLP, where CLP occurs in association with non‐cleft clinical features, allied to developmental studies using appropriate animal models is central to our understanding of the molecular events underlying development of the lip and palate and, ultimately, how these are disturbed in CLP.

## 1 MORPHOGENESIS OF THE LIP AND PALATE

Development of the lip and palate involves a complex series of events that requires close co‐ordination of cell migration, growth, differentiation and apoptosis (Dixon et al., 2011; Mossey et al., 2009). During early embryogenesis, neural crest cells delaminate from the neural folds, undergo an epithelial‐mesenchymal transformation and migrate into the developing craniofacial region. Together with ectodermal and mesodermal cells, the neural crest cell‐derived ecto‐mesenchymal cells participate in formation of the branchial arches and facial processes (reviewed in Compagnucci et al., 2021).

**FIGURE 1:**
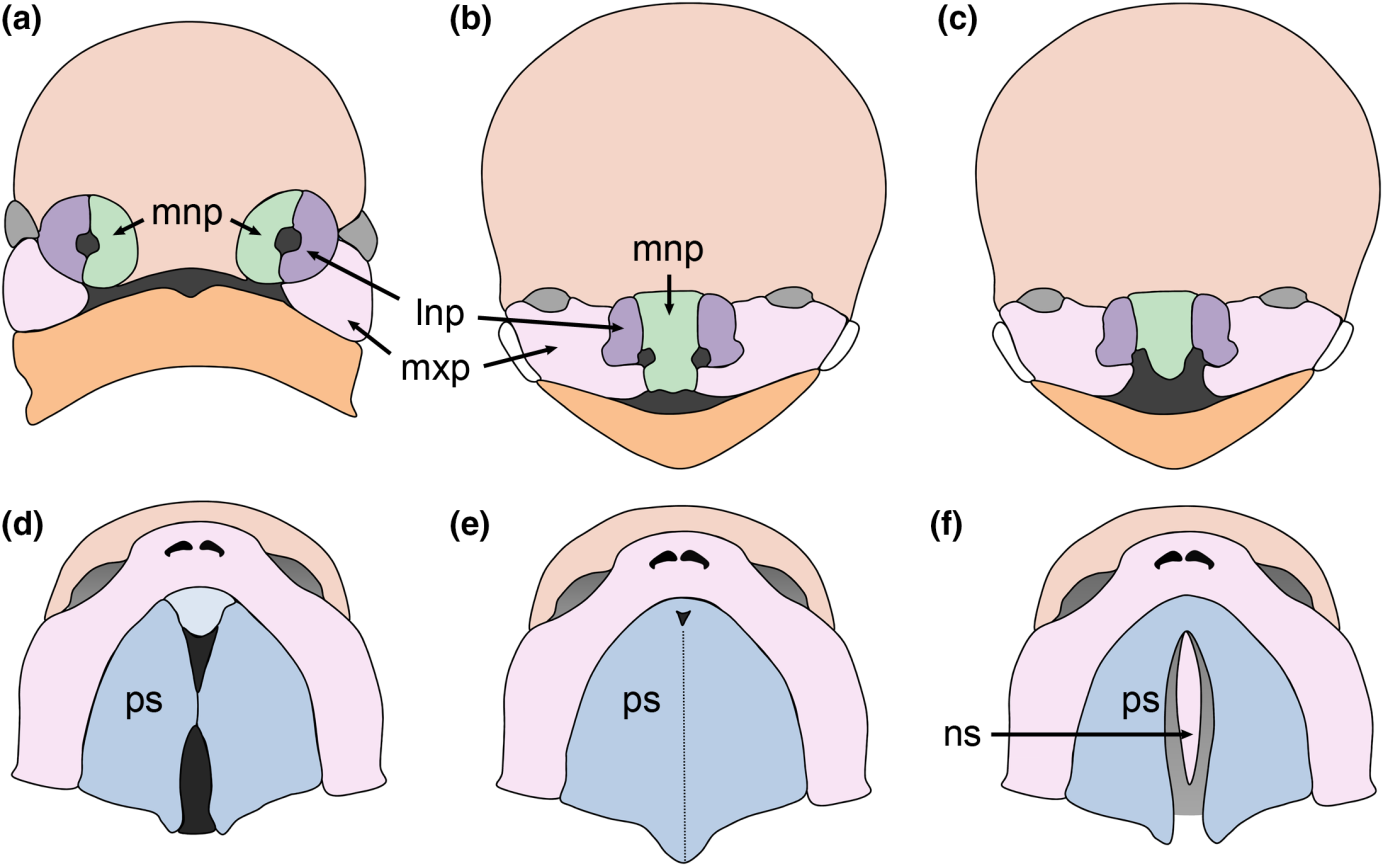
Human facial development. (a) Migratory neural crest cells populate the facial processes. (b) The medial and lateral nasal processes (mnp & lnp, respectively) fuse with the maxillary processes (mxp) to form the upper lip. (c) Bilateral cleft lip. (d) The secondary palate develops from the maxillary processes. (e) The paired palatal shelves (ps) grow vertically before elevating to a horizontal position above the tongue and fusing via the midline epithelial seam. Subsequently, the palatal mesenchyme differentiates into bone and muscle forming the hard and soft palate, respectively. (f) Failure of these processes results in cleft palate with the nasal septum (ns) visible

By the fourth week of human embryogenesis, the frontonasal, maxillary and mandibular prominences surround the oral cavity. Formation of the nasal placodes divides the frontonasal prominence into paired medial and lateral nasal processes (Figure 1a). During the sixth week of embryonic development, the medial nasal processes fuse with the maxillary and the lateral nasal processes to form the upper lip (Figure 1b). The medial nasal processes subsequently fuse together in the midline to form the inter‐maxillary segment which creates the philtrum of the upper lip and the primary palate (Figure 1b). The lateral nasal processes eventually form the alae of the nose while the medial nasal processes and the maxillary processes form the upper lip. Failure of these events results in cleft lip (Figure 1c). The lower lip and lower jaw are formed as a result of midline fusion of the mandibular processes, while lateral fusion of the mandibular and maxillary processes forms the commissures of the mouth.

The secondary palate develops as bilateral outgrowths from the maxillary processes, the palatal shelves initially growing vertically behind the primary palate and lateral to the developing tongue. The palatal shelves re‐orientate above the tongue during the eighth week of gestation, elevation occurring first in the anterior region of the palate before progressing posteriorly (Burdi & Faist, 1967). Subsequently, the palatal shelves grow towards the midline and contact each other. The medial edge epithelia (MEE) at the tips of the palatal shelves adhere and fuse to form a midline epithelial seam (MES). Contact and fusion occur first in the anterior third of the palate before progressing anteriorly and posteriorly during the ninth week of gestation (Figure 1d) (Burdi & Faist, 1967). Consequently, the anterior region of the secondary palate fuses with the primary palate. Degeneration of the MES results in mesenchymal continuity across the palate. The palatal mesenchyme differentiates into bone and muscle to form the hard and soft palate, respectively. These processes are complete by the tenth week of embryogenesis and divide the oronasal space into separate oral and nasal cavities (Figure 1e); a process that is necessary for simultaneous breathing and feeding (Gritli‐Linde, 2007). Failure of these processes results in cleft palate (Figure 1f).

While the descriptive embryology of facial development is established, the underlying molecular mechanisms are only partially characterised. This lack of knowledge represents a major hurdle for understanding how disruption of embryological events results in CLP; consequently, numerous studies have been directed towards increasing our understanding of the molecular events underpinning development of the lip and palate and how these are disturbed in CLP.

The lack of human embryonic tissue and the accompanying ethical issues has resulted in most studies of facial development being performed using animal models such as mice, chicks and zebrafish. As the mouse genome can be manipulated genetically and accurately staged embryos are readily available, the mouse is the main model organism used in the study of facial development and will be the focus of this review.

**FIGURE 2:**
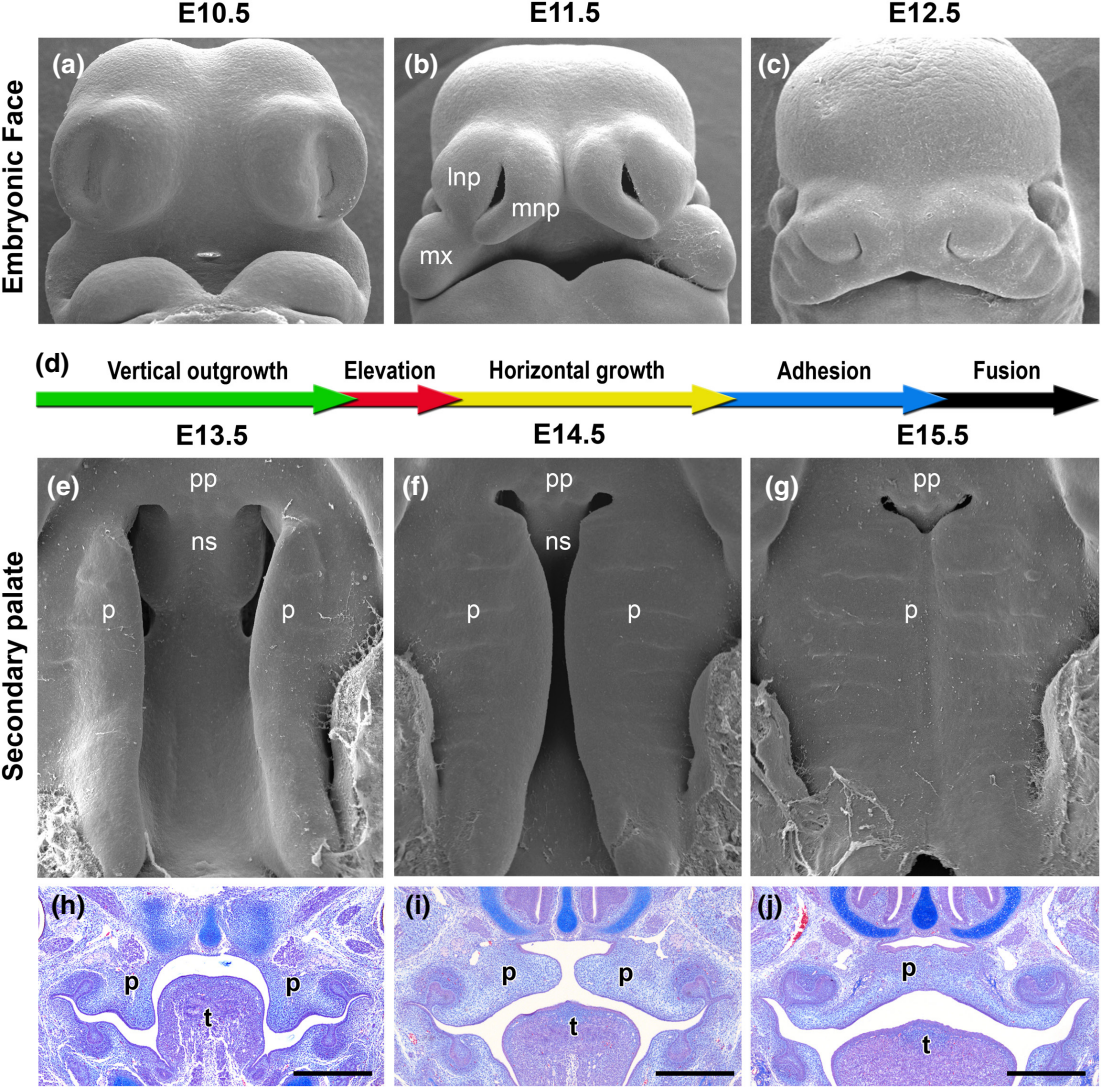
Development of the lip and palate in mice. (a‐c: scanning electron microscopy ‐ frontal views) The upper lip and primary palate form from a series of facial processes which merge by E12.5. (d) Timeline of secondary palate development. (e–g: scanning electron microscopy ‐ ventral views; h–j: histological analysis) The palatal shelves develop from the maxillary processes and grow vertically lateral to the tongue during E12 and E13 (e, h). (f, i) During E14, the palatal shelves elevate above the tongue and fuse in the midline via the midline epithelial seam (g, j). mnp: medial nasal processes; lnp, lateral nasal processes; mx, maxillary processes; pp, primary palate; p, palatal shelves; t, tongue

Development of the mouse lip and palate is similar to that of humans (Figure 2). Lip/primary palate formation commences on embryonic day (E) 9.5, the nasal pits dividing the frontonasal prominence into medial and lateral nasal processes by E10.5 (Figure 2). Merging of the medial nasal, lateral nasal and maxillary processes at the three‐way epithelial seam named the lambdoid junction (λ) at E11.5 ensures that the upper lip is continuous by E12.5. The palatal shelves initiate from the maxillary processes and grow vertically throughout E12 and E13. During E14, the palatal shelves rapidly elevate above the tongue, contact and fuse to form the MES at E14.5, which degenerates by E15.5 (Figure 2).

## 2 SPECIFICATION AND OUTGROWTH OF THE FACIAL PROCESSES

The frontonasal prominence forms as the result of migration of cranial neural crest cells from the fore‐ and mid‐brain regions, migration of cranial neural crest cells from more caudal regions of the mid‐brain and the hind‐brain contributing to formation of the first branchial arch (Osumi‐Yamashita et al., 1994). While the molecular signals controlling the identity of each facial process is unclear, highly conserved gene expression patterns are ultimately responsible for patterning different regions of the head and neck (Cobourne, 2000). Although *Hox* genes control patterning of the branchial arches, it is unlikely that they play a role in establishing the identity of specific facial processes within the first branchial arch as they are not expressed in this territory (Hunt & Krumlauf, 1991). Thus, research has endeavoured to identify other molecules that may establish the positional fate of the migrating cranial neural crest cells and control patterning of the first branchial arch.

Fibroblast growth factor 8 (FGF8), a signalling peptide which is expressed in the oral ectoderm of the first branchial arch in the mouse from E9.5, has been proposed to be responsible for establishing rostral‐caudal polarity of the first branchial arch (Tucker et al., 1999). Early cranial neural crest cell‐derived ecto‐mesenchymal cells respond differently depending on their proximity to the FGF8 signal (Tucker et al., 1999). After the rostral‐caudal axis has been established, polarity is maintained by the combined action of FGF8 and endothelin‐1 (Et‐1) (Tucker et al., 1999).

The genomically linked, distal‐less homeobox (*Dlx*) genes play a central role in specifying the identity of the mandibular and maxillary processes (Depew et al., 2002). Initially, *Dlx* genes are expressed in overlapping patterns in the first branchial arch. Subsequently, the mandibular processes express *Dlx1*, *Dlx2*, *Dlx3*, *Dlx7*, *Dlx5* and *Dlx6*, while the maxillary process expresses only *Dlx1* and *Dlx2* (Qiu et al., 1997) with the mandible undergoing a homeotic transformation into a second maxilla in *Dlx5* ^−/−^; *Dlx6* ^−/−^ mice (Depew et al., 2002).

After their specification, the facial processes grow, approximate and subsequently fuse to prevent cleft lip. At this stage, each facial process consists of a core of ecto‐mesenchyme surrounded by a bi‐layered epithelium comprising cuboidal, basal epithelial cells and a superficial layer of elongated, flattened peridermal cells (Thomason et al., 2008). The epithelium provides a source of growth factors, including FGFs, bone morphogenetic proteins (BMPs) and Sonic Hedgehog (SHH), which control proliferation and apoptosis of the underlying mesenchyme (Hu & Helms, 1999; Richman & Lee, 2003; Richman & Tickle, 1989). If the epithelium is removed, outgrowth and patterning of the facial processes are impaired (Hu & Helms, 1999).

Sonic Hedgehog is a secreted protein which signals to recipient cells by binding to the Patched receptors, PTCH1 and PTCH2. In the absence of ligand binding, PTCH1/PTCH2 inhibit Smoothened (SMO), an obligatory component of the Hedgehog (Hh) signalling pathway. *Smo* activation regulates target gene expression via the GLI transcription factors: GLI1 functions as an activator, while GLI2 and GLI3 act as activators or repressors (McMahon et al., 2003). Although the premature lethality of embryos lacking *Shh*, *Ptch1* or *Smo* initially precluded analysis of SHH function in facial development (Chiang et al., 1996; Goodrich et al., 1997; Zhang et al., 2001), manipulation of Hh responsiveness via conditional gene targeting of *Smo* in neural crest cells allowed the role of Hh signalling in the craniofacial complex to be analysed (Jeong et al., 2004). Although initial formation of the branchial arches was normal, expression of several *Fox* genes, including *Foxc2*, *Foxd1*, *Foxd2*, *Foxf1* and *Foxf2*, was lost resulting in increased apoptosis and decreased cellular proliferation in the frontonasal prominence and mandibular processes leading to facial truncation (Jeong et al., 2004). In contrast, activation of Hh signalling within neural crest cells led to hyperplasia of the facial processes (Jeong et al., 2004). Excess SHH signalling in the facial processes of the chick embryo also resulted in a widening of the frontonasal prominence and, in its most severe form, duplication of facial structures (Hu & Helms, 1999). Recently, inhibition of SHH signalling by administration of cyclopamine to pregnant mice for a 24‐h window commencing on E8.25 resulted in deficiency of the medial nasal processes which prevented contact and fusion with the maxillary processes (Everson et al., 2017). In this model of cleft lip, transcriptional profiling indicated that SHH pathway activity and *Foxf2* expression corresponded with reduced mesenchymal cell proliferation in the medial nasal processes with *Foxf2* being demonstrated to be a direct target of Shh signalling (Everson et al., 2017).

Further support for the importance of SHH signalling during facial growth has been provided through manipulation of *Hhat*, which encodes an acyltransferase responsible for modification of Hh proteins and *Ptch1* (Kurosaka et al., 2014). Mice carrying compound mutations in *Hhat* and *Ptch1* exhibited perturbations in the SHH gradient during development of the frontonasal process which led to hypoplasia of the medial and lateral nasal processes and CLP (Kurosaka et al., 2014).

Bone morphogenetic proteins are also a group of signalling molecules that regulate developmental processes including cellular proliferation, cell death and differentiation. *Bmp2* and *Bmp4* are expressed in restricted domains in the ectoderm covering the distal aspects of the chick facial processes in a pattern that correlates with expression of the transcription factors *Msx1* and *Msx2* in the underlying mesenchyme (Barlow & Francis‐West, 1997; Francis‐West et al., 1994). Ectopic application of BMP2 or BMP4 activated *Msx1*/*Msx2* expression and resulted in overgrowth and altered patterning of the developing facial primordia in chicks (Barlow & Francis‐West, 1997). Similarly, implantation of the BMP antagonist Noggin into the facial processes of the developing chick led to reduced proliferation and outgrowth of the frontonasal prominence and maxillary processes and, ultimately, to deletion of the maxillary and palatine bones (Ashique et al., 2002).

Mutations in the gene encoding the transcription factor p63 underlie a series of human congenital anomalies, a subset of which exhibit CLP as a defining feature (Bokhoven & Brunner, 2002). The *TP63* gene encodes at least six protein variants. Different promoters produce two alternative N‐termini: TA‐isoforms which contain a transactivation sequence; and ∆N‐isoforms which possess an activation domain (Bokhoven & Brunner, 2002). Both isoforms undergo alternative splicing towards the C‐terminus minimally giving rise to α, β and γ variants (Bokhoven & Brunner, 2002). All isoforms contain a DNA‐binding domain, but they vary in their ability to activate or repress their target genes (Ghioni et al., 2002; Yang et al., 1998). In mice, loss of ΔNp63α function recapitulates the CLP phenotype observed in humans (Romano et al., 2012; Thomason et al., 2008). Analysis of *Tp63* ^−/−^ mice revealed defects in mesenchymal cell proliferation in specific regions of the medial nasal, lateral nasal and maxillary processes which resulted in changes in their morphology thereby preventing contact and subsequent fusion (Thomason et al., 2008). Analysis of key signalling molecules revealed increased *Bmp4* while *Shh* and *Fgf8* expression were downregulated in the epithelia of the facial processes of *Tp63* ^−/−^ embryos in areas overlying those regions in which decreased mesenchymal cell proliferation occurred (Thomason et al., 2008). Notably, ΔNp63α also regulates WNT signalling and cell adhesion molecules during ectodermal development (Fan et al., 2018; Ferone et al., 2013).

## 3 ADHESION AND FUSION OF THE FACIAL PROCESSES

As the facial processes approximate, filopodia span out from the epithelia to facilitate the initial contact and fusion of the apposed cells; in the chick cleft primary palate (*cpp*) mutant filopodia are absent and the facial processes fail to fuse (Cox, 2004).

Candidate genes for molecules that play a central role in initial adherence of the epithelia covering the facial processes include nectin1 and E‐cadherin. Nectin1 is an immunoglobulin‐type, cell‐cell adhesion molecule that participates in the formation of adherens junctions with E‐cadherin. The *PVRL1* gene, which encodes nectin1, is mutated in patients with CLP and ectodermal dysplasia 1 (CLPED1) (Suzuki et al., 2000). Nevertheless, despite being highly expressed in the epithelia of the facial processes during mouse embryonic development (Cox, 2004), CLP is not observed in *Pvrl1* knockout mice; rather they display defects in dental enamel formation (Barron et al., 2008; Inagaki et al., 2005). This observation suggests that, at least in mice, other nectins or adhesion molecules can compensate for the loss of nectin1.

Similarly, although mutations in CDH1, the gene encoding E‐cadherin, underlie a subset of cases of CLP in humans (Ghoumid et al., 2017), *Cdh1*‐null mice die during early embryogenesis (Riethmacher et al., 1995). Despite these observations, a recent study has demonstrated that mutations in genes encoding members of the epithelial cadherin‐p120‐catenin complex, including *CTNND1*, *PLEKHA7*, *PLEKHA5* and *CDH1*, and the epithelial splicing regulator *ESRP2* underlie a subset of cases of Mendelian non‐syndromic CLP (Cox et al., 2018). Moreover, mice in which *Ctnnd1* is ablated in the developing oral epithelia display a full spectrum of CLP phenotypes ranging from overt clefts to delayed growth of the maxillary processes (Cox et al., 2018). Similarly, while *Esrp2* knockout mice appear phenotypically normal, mice lacking *Esrp1* function display fully penetrant bilateral CLP; however, *Esrp2* can partially compensate for loss of *Esrp1*, as the facial phenotypes observed in *Esrp1*‐null embryos are more severe in combination with deletion of one or both *Esrp2* alleles (Bebee et al., 2015). Further support for *Esrp2* playing an important role in facial development has been provided by the recent suggestion that a frameshift mutation in this gene is likely to be responsible for the *cpp* phenotype in chicks (Youngworth & Delany, 2020).

Phenotypic analysis of the facial processes of *Esrp1*‐null mice showed decreased proliferation in epithelial and mesenchymal cells of both the medial and lateral nasal processes resulting in reduced growth of the facial processes and a failure to fuse (Lee et al., 2020). RNA‐Seq analysis subsequently identified large‐scale changes in the splicing of genes expressed in the ectoderm, including *Fgfr2* and *Ctnnd1*, as well as changes in total transcript levels in *Esrp1* ^−/−^ ectoderm compared with that of wild‐type embryos (Lee et al., 2020). The latter group of genes included decreases in expression of canonical WNTs, including *Wnt9b* and *Shh*, components of two pathways that are essential for lip and/or palate development (Everson et al., 2017; Jin et al., 2012). Notably, these alterations were associated with corresponding down‐regulation of canonical WNT and SHH‐regulated targets, including *Gli* genes, *Foxf1*, *Foxf2* and *Osr2*, in the subjacent mesenchyme, suggesting that a reduction in signalling from ectoderm to mesenchyme leads to reduced mesenchymal proliferation (Lee et al., 2020).

Wingless‐related integration site (WNT) signalling molecules are secretory glycoproteins which signal through Frizzled (FZD) cell surface receptors. WNT signalling activates signal transduction pathways which control cell proliferation, cell polarity, cell differentiation and cell survival (Smalley & Dale, 1999). *Wnt* genes and canonical WNT signalling reporter transgenes are expressed at high levels in the mouse facial prominences and their derivatives, supporting a potential role of WNT/β‐catenin signalling during facial development (Geetha‐Loganathan et al., 2009; Lan et al., 2006; Maretto et al., 2003; Summerhurst et al., 2008). Analyses of genetically altered mice in which key components of the WNT signalling pathway are disrupted have provided further support for this hypothesis. For example, ablation of the canonical WNT signalling co‐receptor gene *Lrp6* results in decreased expression of the WNT target genes *Msx1* and *Msx2*, reduced cell proliferation and hypoplasia of the facial processes leading to CLP in mice (Song et al., 2009). Similarly, inactivation or constitutive activation of the gene encoding β‐catenin, *Ctnnb1*, in the embryonic facial ectoderm result in down‐regulation or upregulation, respectively, of ectodermal FGF family gene expression and severe facial defects (Reid et al., 2011; Wang et al., 2011).

## 4 FUSION OF THE FACIAL PROCESSES

After initial contact and adhesion, the epithelia covering the facial processes fuse together to form an epithelial seam at λ; this is subsequently removed to allow mesenchymal confluence. Transformation of epithelial cells into mesenchymal cells and apoptosis of the epithelial seam have been proposed to by the main mechanisms by which the epithelial cells are eliminated (Jiang et al., 2006; Sun et al., 2000). In the chick, the outer periderm cells undergo apoptosis and slough from the facial processes prior to contact so that the basal epithelial cells make direct contact (Sun et al., 2000), a process that has also been observed in human embryos (Hinrichsen, 1985). Recently, a substantial number of epithelial cells within the epithelial seam have also been demonstrated to undergo apoptosis (Jiang et al., 2006).

**FIGURE 3:**
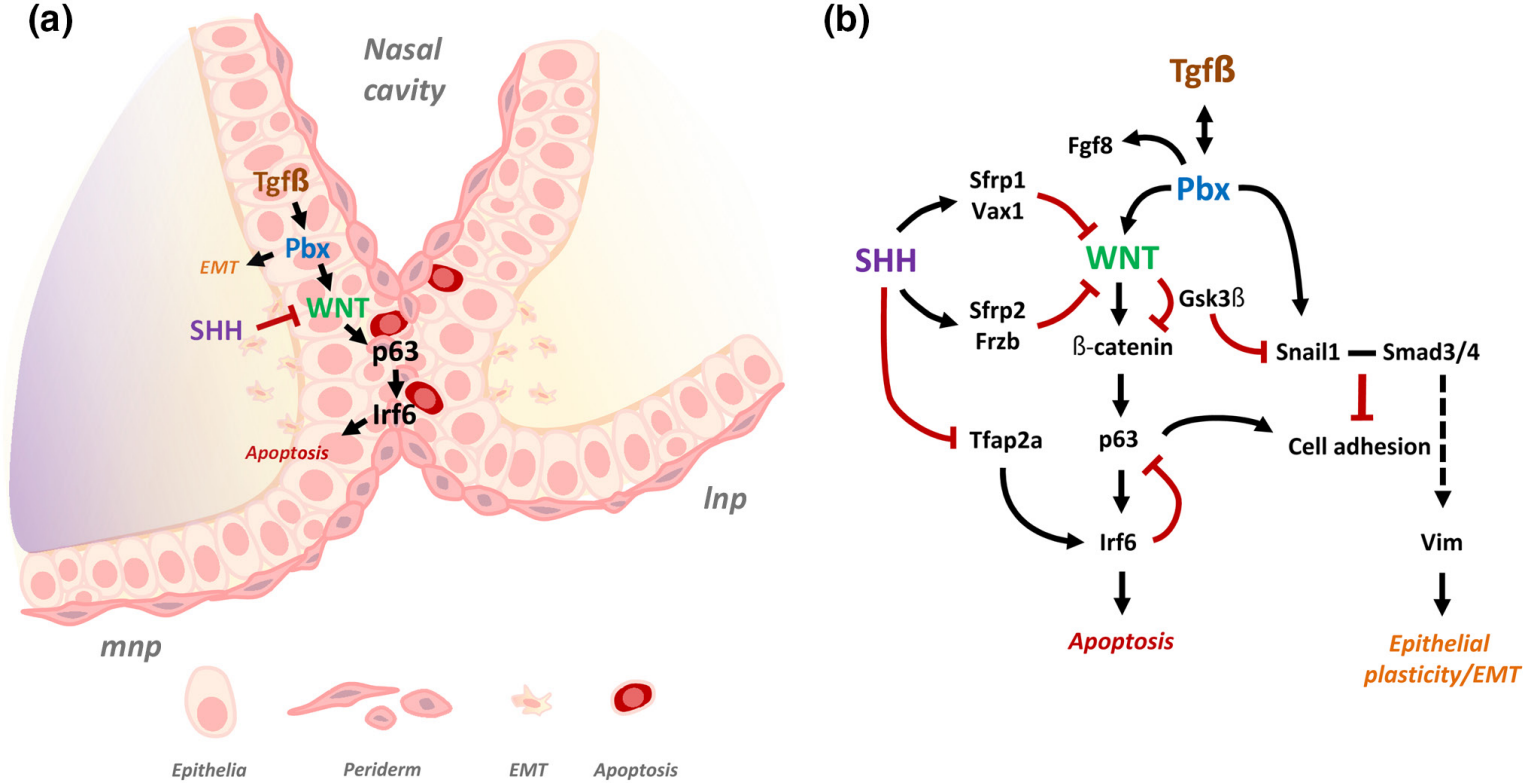
Molecular mechanisms of lip fusion and epithelial seam dissolution. (a) Schematic diagram of the lambdoidal (λ) epithelial seam at E11.5, formed through fusion of the medial nasal and lateral nasal processes. SHH and TGFβ‐mediated Pbx signalling converge on WNT to regulate pathways involved in epithelial seam dissolution. (b) Pbx plays a dual‐role in lip fusion, (1) by regulating a WNT‐p63‐Irf6 cascade to promote epithelial apoptosis; (2) by promoting epithelial‐mesenchymal transformation, cell plasticity/migration through regulation of Snail1. Cross‐talk between both pathways is achieved by post‐translational modification of Gsk3β on Snail1. SHH ensures appropriate p63‐Irf6 signalling by up‐regulating WNT antagonists and restricting Tfap2a signalling. (Adapted from Kurosaka et al., 2014; Losa et al., 2018). mnp, medial nasal process; lnp, lateral nasal process; EMT, epithelial‐mesenchymal transformation

At the molecular level, a *Pbx*‐*Wnt*‐*Tp63*‐*Irf6* regulatory network controls facial morphogenesis by promoting epithelial apoptosis at λ (Figure 3) (Ferretti et al., 2011). Building on the observations that mutations in the genes encoding WNT3, IRF6 and TP63 lead to CLP in humans (Celli et al., 1999; Kondo et al., 2002; McGrath et al., 2001; Niemann et al., 2004), Ferretti and co‐workers demonstrated that PBX proteins control WNT signalling directly by binding to an enhancer that drives *Wnt9b* and *Wnt3* expression in the midface (Ferretti et al., 2011). In turn, WNT signalling activates *Tp63* expression *via* Lef‐Tcf binding to a conserved regulatory element with p63 subsequently activating *Irf6* expression by enhancer binding (Ferretti et al., 2011). Notably, expression of *Fgf8*, but not that of *Fgf9*, was also downregulated at λ of E10.5‐E11.5 *Pbx* mutant and *Wnt9b* ^−/−^ mice, integrating FGF signalling into the PBX controlled regulatory network (Ferretti et al., 2011). Subsequently, the same group demonstrated that cell death alone is insufficient to remove the epithelial seam at λ and that PBX‐dependent transcriptional regulation of the epithelial‐mesenchymal transformation driver Snail1 also played an important role (Figure 3) (Losa et al., 2018).

In addition to its importance in their growth, SHH signalling plays an important role in removal of the epithelial seam by restricting canonical WNT signalling during fusion of the facial processes; this is achieved through stimulating expression of genes encoding WNT inhibitors including *Sfrps*, *Frzbs* and *Vax1* (Figure 3) (Kurosaka et al., 2014). In turn, this cascade allows appropriate expression of *Irf6* and *Tp63* thereby permitting degeneration of the epithelial seam at λ. In addition, *Shh* controls expression of the transcription factor *Tfap2a* in the medial nasal processes (Figure 3). In this context, mutations in *TFAP2A* underlie branchio‐oto‐renal syndrome in which CLP is a defining feature (Milunsky et al., 2008) and disruption of a *TFAP2A*‐binding site by the risk allele of rs642961 in an *IRF6* enhancer contributes to non‐syndromic CLP (Rahimov et al., 2008).

Along with its role in growth of the facial processes, *Bmp4* is involved in degeneration of the epithelial seam at λ. In the mouse, *Bmp4* expression is restricted to the region of epithelia within the facial processes that contact and fuse. This region corresponds to that in which NOGGIN expression is absent in the developing chick face (Ashique et al., 2002; Gong & Guo, 2003). After fusion, *Bmp4* expression switches to the underlying mesenchyme. It has been suggested that *Bmp4* may mediate apoptosis of the periderm cells within the region of fusion (Gong & Guo, 2003). Conditional inactivation of the Bmp receptor‐1a (*Bmpr1a*) gene in the epithelia of the facial processes resulted in fully penetrant bilateral CLP. In contrast, deletion of *Bmp4* from the same region led to isolated cleft lip (Liu et al., 2005).

Recently, in an elegant high‐resolution transcriptomic analysis of fusion of the upper lip and primary palate, Li and co‐workers used single‐cell RNA sequence analysis to examine the molecular anatomy of the E11.5 mouse λ junction (Li et al., 2019a, 2019b). This study defined numerous cell populations that contribute to fusion of the facial processes with the data providing a powerful resource that will inform future studies of the molecular events driving this important developmental process and how they are disturbed in cleft lip.

## 5 SPECIFICATION AND OUTGROWTH OF THE PALATAL SHELVES

**FIGURE 4:**
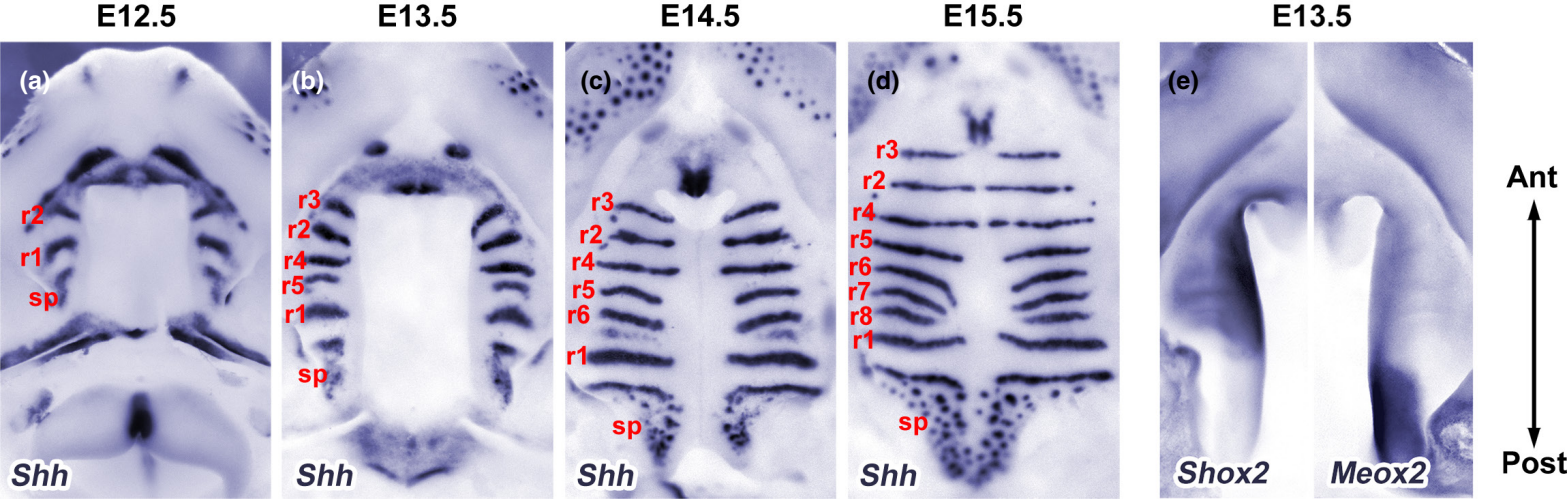
Sequential rugae interposition during secondary palate growth and patterning. (a–e) Whole‐mount in situ hybridisation of E12.5‐E15.5 palatal shelves. (a–d) During palatogenesis, *Shh* expression defines epithelial rugae (r1‐r8) and their sequential interposition on the oral surface. In the posterior palate, Shh is expressed in sensory papillae. (e) *Shox2* (anterior) and *Meox2* (posterior) show differential gene expression along the anterior‐posterior axis, the boundary of which is defined by R1. r, ruga; sp, sensory papillae

Although the signal that initiates palatal outgrowth is unknown (Bush & Jiang, 2012), SHH signalling plays an important role in growth and patterning of the palatal shelves. Prior to palatal outgrowth, *Shh* is expressed throughout the oral epithelium (Jeong et al., 2004; Rice et al., 2004) subsequently becoming restricted to the rugae, which are a series of transverse epithelial thickenings in the anterior palate, and the sensory papillae in the posterior palate (Figure 4) (Pantalacci et al., 2008; Rice et al., 2006; Welsh & O'Brien, 2009). Rugae form in a defined periodic sequence during anterior palatal extension through a Turing‐type reaction‐diffusion mechanism (Economou et al., 2012; Pantalacci et al., 2008; Welsh & O'Brien, 2009). Formation of the first ruga defines the boundary of the future hard and soft palate, with sequential rugae interposition occurring in the rugal growth zone which lies anterior to this landmark (Figure 4) (Pantalacci et al., 2008; Welsh & O'Brien, 2009).

Gene expression studies have revealed considerable molecular heterogeneity along both the anteroposterior and oronasal axes of the palatal shelves (reviewed by Bush & Jiang, 2012; Hilliard et al., 2005; Lan et al., 2015; Li et al., 2017a, 2017b). For example, expression of the transcription factors *Msx1* and *Shox2* is restricted to the anterior palate, while *Meox2* and *Tbx22* are confined to the posterior palate (Figure 4). These factors are intrinsic to regional growth of the palate such that *Shox2* ^−/−^ mice exhibit rare incomplete clefting of the anterior palate (Yu et al., 2005) while *Tbx22*, which is regulated by *Mn1*, is required for posterior palatal growth (Liu et al., 2008). Furthermore, complex networks of genes are associated with rugal (e.g., *Shh*, *Spry2*, *Hes1*) or inter‐rugal zones (e.g., *Fgfr2*, *Etv5*, *Sostdc1*) on the oral side of the palate (Welsh & O'Brien, 2009) which are distinct from the genes expressed on the nasal aspect, reflecting cell fate differences within the palate (Hammond et al., 2018; Han et al., 2009; Hilliard et al., 2005). These studies highlight the spatial heterogeneity of the epithelium and mesenchyme during secondary palate development and imply complex cross‐talk between multiple molecular pathways.

The gene encoding the SHH receptor, *Ptch1*, is expressed both in rugae and the adjacent mesenchyme, whereas the transmembrane transducer of SHH signalling, *Smo*, is expressed in the palatal mesenchyme (Rice et al., 2006). Mice with targeted *Shh*‐null or cranial neural crest‐specific (*Wnt1*‐Cre) deletion of *Smo*, display severe early craniofacial abnormalities which precluded analysis of secondary palate development (Chiang et al., 1996; Jeong et al., 2004). However, studies investigating tissue‐specific deletion of *Shh* in the palatal epithelium using *Krt14*‐Cre (Rice et al., 2004) or *Smo* in the palatal mesenchyme using *Osr2*‐Cre (Lan & Jiang, 2009) confirmed that SHH signals from the epithelium to the mesenchyme to control palatal cell proliferation and outgrowth (Lan & Jiang, 2009; Rice et al., 2004). In addition, mice with loss or gain of *Smo* function within the palatal mesenchyme exhibit cleft palate, demonstrating that precise control of SHH‐SMO signalling is required for normal palate development (Hammond et al., 2018; Lan & Jiang, 2009).

**FIGURE 5:**
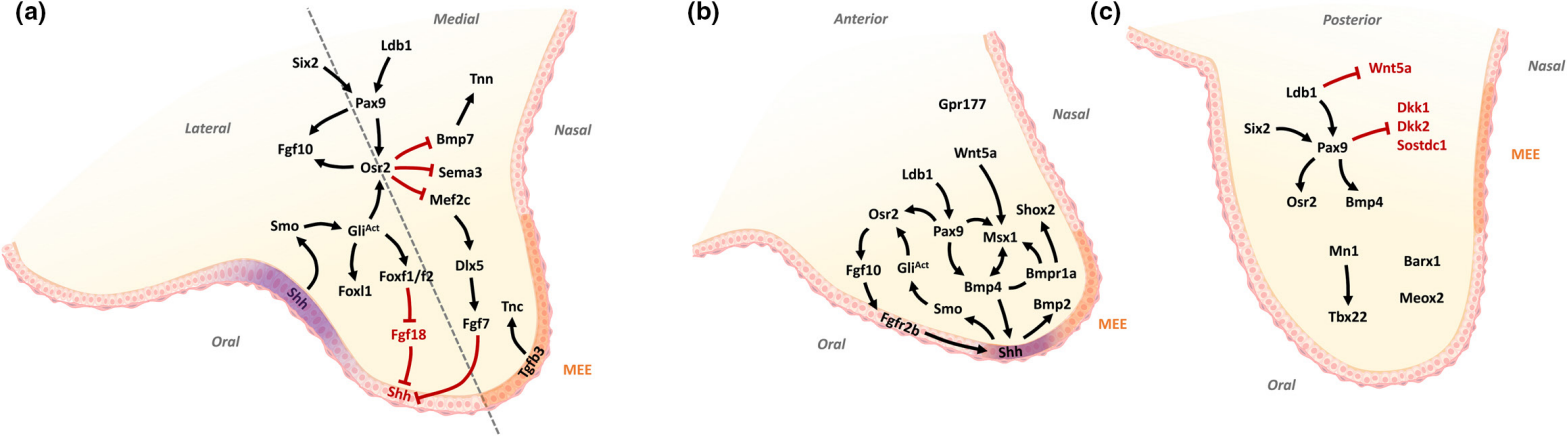
Molecular regulation of secondary palate growth and patterning. (a, b) Schematic diagram showing molecular regulation and cross‐talk of key molecules involved in secondary palate growth and patterning. (a, b) *Shh* is a key gene in oral‐nasal patterning, signalling from the oral epithelium to the underlying mesenchyme to positively regulate *Osr2*, *Fgf10* and *Foxf1*/*Foxf2*/*Foxl1* molecular cascades. *Fgf10* and *Bmp4* maintain expression of *Shh*, whereas *Dlx5*‐*Fgf7* signalling in the nasal mesenchyme restricts *Shh*. (b) *Shox2* and *Msx1* expression is restricted to the anterior palate. Outgrowth of the anterior palate is controlled by a network involving *Shh*, *Bmp*, *Msx1*, *Wnt5a* and *Pax9*. (c) *Meox2*, *Barx1* and *Tbx22* expression is restricted to the posterior palate. *Pax9* has a central role in regulating posterior signalling, promoting *Osr2*, *Bmp4* and WNT signalling. Adapted from Li et al., 2017. MEE, medial edge epithelia

Fibroblast growth factor signalling is also essential for outgrowth and patterning of the secondary palate, with multiple members of the FGF family converging on the regulation of *Shh*. *Fgf10* is expressed in the palatal mesenchyme while its receptor, *Fgfr2b*, is expressed in the overlying inter‐rugal epithelium (Rice et al., 2004; Welsh & O'Brien, 2009). *Fgf10* ^−/−^ and *Fgfr2b* ^−/−^ embryos exhibit cleft palate with impaired cellular proliferation, reduced outgrowth and diminished *Shh* expression (Rice et al., 2004). Similarly, mesenchymal expression of *Fgf10* is dependent on SHH‐SMO signalling (Lan & Jiang, 2009). Collectively, these studies demonstrate that SHH and FGF10 function in a positive feedback loop to regulate palatal outgrowth via reciprocal epithelial‐mesenchymal interactions (Lan & Jiang, 2009; Rice et al., 2004). Conversely, *Fgf7* is expressed in a complementary pattern in future nasal mesenchyme prior to palatal shelf elevation (Veistinen et al., 2009) and is regulated by *Dlx5* which functions antagonistically to restrict *Shh* to the oral epithelium (Han et al., 2009). Furthermore, palatal explant cultures demonstrated that exogenous FGF10 or FGF7 induced or repressed epithelial *Shh*, respectively, while exogenous SHH applied to mesenchymal explants repressed *Fgf7* (Han et al., 2009; Rice et al., 2004). However, the palate develops normally in *Fgf7* ^−/−^ mice indicating that other signalling molecules act downstream of *Dlx5* (Guo et al., 1996). Together these studies demonstrate that multiple signalling pathways converge on SHH signalling, which plays a central role in oronasal patterning of the secondary palate (Figure 5).

SHH‐SMO signalling positively regulates the expression of several *Fox* transcription factors in the cranial neural crest cells that populate the developing facial processes (Jeong et al., 2004). Recently, modulation of *Smo* within the palatal mesenchyme revealed *Foxd1*, *Foxd2*, *Foxf1*, *Foxf2*, *Foxl1* and *Foxq1* are dependent on SHH‐SMO signalling (Hammond et al., 2018; Lan & Jiang, 2009), with *Foxf1*, *Foxf2 and Foxl1* being expressed in overlapping domains within the oral palatal mesenchyme underlying the rugae (Hammond et al., 2018; Lan & Jiang, 2009). In addition, *Foxl1* was shown to be a direct target of the Shh transcriptional activator Gli1 (Hammond et al., 2018). Although *Foxf1* ^−/−^ mice exhibit embryonic lethality prior to craniofacial development (Mahlapuu et al., 2001), *Foxf2* ^−/−^ mice exhibit cleft palate, with intrinsic defects in palatal shelf growth, extracellular matrix composition and loss of epithelial *Shh* (Nik et al., 2016; Xu et al., 2016). Analyses of *Foxf2* ^−/−^ mice have also revealed ectopic expression of *Fgf18* in regions where *Foxf2* is normally expressed. Furthermore, palatal explant cultures treated with exogenous FGF18 inhibited *Shh* expression in the epithelium, demonstrating a novel *Shh*‐*Foxf*‐*Fgf18*‐*Shh* circuit during palate development (Xu et al., 2016).

Recently, an integrative genome‐wide analysis indicated that *Foxf2* directly represses several transcription factors involved in extracellular matrix organisation and osteogenesis in the developing palate. As noted above, several targets were upregulated or ectopically expressed in *Foxf2* ^−/−^ mice, including *Fgf18* (Xu et al., 2016) *Foxq1* and *Exoc2*, indicating that a major role of *Foxf2* is to repress or prevent aberrant gene expression (Xu et al., 2020). Subsequently, an elegant study revealed that *loxP*‐targeted deletion of *Foxf2* also disrupted flanking genomic regions that included the lncRNA *1700018A04Rik*, revealing novel *cis*‐regulation of *Foxq1* and *Exoc2* within the same chromatin interaction domain (Xu et al., 2021). This study demonstrated that *cis*‐regulatory enhancers flanking *Foxf2* and/or *1700018A04Rik* are directly involved in *Foxq1*/*Exoc2* repression. Interestingly, *Foxf2*, *Foxq1* and *Exoc2* were all significantly up‐regulated in conditional *Smo* ^+/M2^ gain‐of‐function mice, indicating these genes are targets of SHH‐SMO signalling (Hammond et al., 2018); however, whether these genes contribute to the pathogenesis of cleft palate in *Foxf2* ^−/−^ or conditional *Smo* ^+/M2^ mice requires further investigation. These studies emphasise the need for careful interpretation of gene regulatory mechanisms in traditional promoter knockout mouse models.

The transcription factor Odd‐skipped‐related 2 is an intrinsic regulator of palatal mesenchymal cell proliferation with *Osr2* ^−/−^ mice displaying cleft palate as a result of defects in palatal shelf growth and elevation (Lan et al., 2004). *Osr2* is expressed in the oral mesenchyme throughout the length of the palate and has been shown to function downstream of SHH‐SMO signalling (Lan & Jiang, 2009) and upstream of *Fgf10* (Zhou et al., 2013). In addition, *Pax9* ^−/−^ (Zhou et al., 2013) and *Ldb1* ^−/−^ (Almaidhan et al., 2014) mice exhibit cleft palate with diminished expression of *Osr2*, indicating a complex network of genes regulate *Osr2* to ensure normal growth and patterning of the developing secondary palate (Figure 5).

Transcriptional profiling and expression analyses of *Osr2* ^−/−^ mice revealed upregulation of genes involved in osteogenesis, including *Mef2c*, *Sox6*, *Sp7* and various BMP ligands (*Bmp3*, *Bmp5* and *Bmp7*) (Fu et al., 2017). Furthermore, several class‐3 semaphorins (*Sema3a*, *Sema3d* and *Sema3e*) were expressed ectopically and shown to be direct targets (*Sema3a* and *Sema3d*) of *Osr2*. Together, these studies reveal OSR2 plays an intrinsic role in mesenchymal cell proliferation and fate, preventing premature osteogenesis and aberrant semaphorin expression. However, further studies are needed to understand the function of semaphorins during palate development (Fu et al., 2017).

As in the primary palate, BMP signalling plays an important role during secondary palate development (reviewed Nie et al., 2006; Parada & Chai, 2012). Ablation of *Bmpr1a* in cranial neural crest cells (Li et al., 2011) or palatal mesenchyme (Baek et al., 2011) resulted in cleft palate with defects in outgrowth of the primary and anterior secondary palate. Similarly, mice with cranial neural crest‐specific deletion of *Acvr1* displayed multiple craniofacial defects including cleft palate and a hypoplastic mandible (Dudas et al., 2004).

Bone morphogenetic protein cross‐talk with SHH signalling has also been demonstrated during secondary palate outgrowth. In the anterior palate, SHH induces *Bmp2* to positively regulate mesenchymal cell proliferation (Zhang et al., 2002). Conversely, deletion of *Smo* in the palatal mesenchyme resulted in downregulation of *Bmp2*, indicating its dependence on SHH‐SMO signalling during secondary palate development (Lan & Jiang, 2009). BMP4 signalling is also crucial to this network with *Msx1* regulating proliferation of the anterior palatal mesenchyme via *Bmp4* expression, which in turn maintains *Shh* expression in the most anterior rugae (Zhang et al., 2002). Interestingly, expression of *Bmp2* and *Bmp4* was increased in mice with a deletion of *Bmpr1a* in the palatal mesenchyme, while *Fgf10* and epithelial *Shh* expression was decreased, indicating that other molecules downstream of BMP signalling is involved in maintaining *Shh* expression (Figure 5) (Baek et al., 2011).

*Bmp4* is expressed in the posterior palate (Levi et al., 2006) where its expression is *Pax9*‐dependent (Zhou et al., 2013). *Pax9* is expressed in a posterior to anterior gradient in the palatal mesenchyme and studies on *Pax9* ^−/−^ mice have revealed cleft palate associated with defects in palatal growth/elevation, while expression of *Bmp4*, *Msx1*, *Fgf10* and *Osr2* was reduced in the palatal mesenchyme. *Pax9* ^−/−^ mice also showed reduced *Shh* expression and disorganised rugae (Zhou et al., 2013). Introduction of *Osr2* into the *Pax9* locus using knock‐in technology was sufficient to restore *Fgf10* expression and partially rescue posterior palate morphogenesis in the absence of *Pax9*; however, *Shh* and *Bmp4* expression were not restored (Zhou et al., 2013). These studies demonstrate that *Pax9* regulates a network involving FGF and BMP signalling targets which converge on *Shh* and *Osr2* signalling (Figure 5).

The transcription factor Sine oculis‐related homeobox 2 (SIX2) is involved in many aspects of early craniofacial development (He et al., 2010a, 2010b; Liu et al., 2019) and was recently shown to play multiple roles during secondary palate development (Okello et al., 2017). Prior to palatal shelf elevation, *Six2* is expressed throughout the palatal mesenchyme. Subsequently, during palatal elevation and fusion, *Six2* is upregulated in the nasal epithelium concomitant with downregulation in the subjacent mesenchyme, suggesting a role in oronasal patterning and cell fate (Okello et al., 2017). Subsequently, *Six2* was shown to play an intrinsic role in mesenchymal cell proliferation (Okello et al., 2017; Sweat et al., 2020), and negatively regulate osteogenesis (Sweat et al., 2020). Phenotypically, *Pax9* ^−/−^ and *Six2* ^−/−^ mice share similar features, and these genes are co‐expressed in the developing palate (Sweat et al., 2020). While *Pax9* is a direct transcriptional target of *Six2* in the palatal mesenchyme (Sweat et al., 2020), further studies are needed to understand the gene regulatory networks controlled by *Six2* during palate development (Figure 5).

WNT signalling plays an important role in *Pax9*‐mediated development of the secondary palate (Jia et al., 2017a, 2017b, 2020; Li et al., 2017a, 2017b). *Axin2* and activated *β‐catenin*, which are direct targets of canonical WNT signalling, were reduced in the posterior palate of *Pax9* ^−/−^ mice correlating with increased expression of the WNT antagonist *Dkk2* (Li et al., 2017a, 2017b). Pharmacological inhibition of DKK activity using the small‐molecule agonists IIIc3a (Li et al., 2017a, 2017b) or WAY‐262611 (Jia et al., 2017b) partly rescued palate morphogenesis and fusion, with only a partial cleft remaining between the primary palate in the anterior region of the secondary palate. Although the dual WNT/BMP antagonist *Sostdc1* was downregulated in *Pax9* ^−/−^ mice, coincident with reduced *Bmp4* expression, genetic inactivation of *Sostdc1* was sufficient to rescue cleft palate and restore canonical WNT signalling in the palatal mesenchyme (Figure 5) (Li et al., 2017a, 2017b).

Ectodysplasin/ectodysplasin A receptor (EDA/EDAR) signalling lies downstream of WNT signalling in other development contexts and *Eda* expression is reduced in *Pax9* ^−/−^ mice (Jia et al., 2017a). Although EDA/EDAR signalling is dispensable for palate formation (Headon & Overbeek, 1999), in utero stimulation of this pathway using an EDAR agonist rescued cleft palate in *Pax9* ^−/−^ mice (Jia et al., 2017a). While treated mice displayed disorganised rugae and expression of *Bmp4*, *Msx1*, *Fgf10* and *Osr2* was not restored, the expression of WNT pathway components was not analysed. Together, these studies suggest that *Pax9* integrates WNT signalling by modulating WNT antagonists in the palatal mesenchyme, but the mechanism underlying transcriptional regulation of WNT target genes requires further investigation.

## 6 PALATAL SHELF ELEVATION AND REMODELLING

Historically, palatal shelf elevation was viewed as a process of rotation (reviewed in Bush & Jiang, 2012); however, an alternative hypothesis of palatal shelf remodelling was subsequently proposed where bulging of the medial aspect occurs simultaneously with retraction of the ventral edge driven by an intrinsic force. Consequently, a combination of rotation of the anterior palate with remodelling of the mid and posterior regions was advanced to explain palatal shelf elevation (Bush & Jiang, 2012), while later studies suggested the palatal shelves ‘flow’ over the tongue (Bush & Jiang, 2012). Whatever the mechanism, this process often occurs asynchronously with one palatal shelf elevating before the other (Bush & Jiang, 2012).

During palatal shelf elevation, the MEE of opposing shelves re‐orientates to enable contact and fusion; hence, the position of the MEE is crucial to understanding this process. While molecular markers including *Tgfb3* and *Mmp13* define the presumptive MEE in the vertical palatal shelves (Blavier et al., 2001; Fitzpatrick et al., 1990; Pelton et al., 1990), their expression has not, until recently, been analysed during palatal shelf remodelling. Analysis of *Zfhx1a* ^−/−^ embryos, which exhibit a delay in palatal shelf elevation, demonstrated that *Mmp13* is expressed from the distal tip to the lingual aspect of the vertical palatal shelves anteriorly, whereas in the mid and posterior regions *Mmp13* localises exclusively on the lingual aspect (Jin et al., 2010). Similarly, extensive remodelling of the palatal mesenchyme has been demonstrated during palatal shelf elevation using region‐specific extracellular matrix markers (Chiquet et al., 2016). Together with a recent histomorphological study, these data confirm that palatal shelf elevation is heterogeneous along the anteroposterior axis (Yu & Ornitz, 2011).

Although the intrinsic forces and molecular mechanisms underlying palatal elevation are incompletely understood, multifunctional components of the extracellular matrix are important for this process (reviewed by Paiva et al., 2019; Wang et al., 2020). Studies have suggested that hyaluronic acid, the predominant glycosaminoglycan of the palatal mesenchyme, generates an intrinsic force that drives palatal shelf elevation (Brinkley & Morris‐Wiman, 1987). Several mouse models with defective palatal shelf elevation have altered expression of glycosaminoglycan/ hyaluronic acid; for example, *Fgfr2^C342Y^* ^/C342Y^ mice exhibit delayed palatal shelf elevation coincident with reduced glycosaminoglycan synthesis (Synder‐Warwick et al., 2010). *Pax9* ^−/−^ mice also display reduced hyaluronic acid accumulation and delayed palatal shelf elevation (Li et al., 2017a, 2017b), while loss of the golgin subfamily b, macrogolgin 1 *Golgb1* results in failure of palatal shelf elevation and increased cell density in the palatal mesenchyme (Lan et al., 2016). Recently, mice with a deletion of exon 2 of *Fgf9* have been shown to exhibit cleft palate and micrognathia, with reduced hyaluronic acid accumulation, increased mesenchymal cell density and delayed palatal shelf elevation (Li et al., 2021).

Hyaluronic acid is synthesised by hyaluronan synthases which are encoded by the genes *Has1*, *Has2* and *Has3*. The genes encoding the hyaluronan synthase enzymes display distinct spatio‐temporal expression patterns during embryogenesis with *Has2*, which is positively regulated by TGFβ3, predominant in craniofacial tissues (Galloway et al., 2013; Tien & Spicer, 2005). Although *Has2*‐null mice exhibit early embryonic lethality, tissue‐specific inactivation of *Has2* in the palatal mesenchyme or cranial neural crest, caused fully penetrant cleft palate resulting from delayed palatal shelf elevation or failed palatal shelf elevation secondary to micrognathia and tongue obstruction, respectively (Lan et al., 2019; Yonemitsu et al., 2020).

Expression studies have revealed that other extracellular matrix components exhibit dynamic expression patterns in the palatal mesenchyme during palatal shelf elevation and closure (Chiquet et al., 2016; Jin et al., 2010). Tenascins are a large family of matricellular proteins that possess the ability to influence cell shape, migration and growth (Chiquet‐Ehrismann, 2004). Members of the tenascin family are differentially expressed during shelf elevation. For example, in mid and posterior regions of the palate, Tenascin‐W (*Tnn*) is restricted to the medial nasal mesenchyme prior to palatal shelf elevation consistent with a role in osteogenesis; in contrast, Tenascin‐C (*Tnc*) is expressed throughout the medial aspect of the palatal mesenchyme (Chiquet et al., 2016). Interestingly, mice with loss or gain of BMP7 signalling exhibit diminished or expanded *Tnn*, respectively, coincident with cleft palate and delayed shelf elevation (d'Amaro et al., 2012; Fu et al., 2017). Together these studies identify *Tnn* as a target of BMP7 signalling and suggest an intrinsic requirement during palatal shelf elevation and reorientation. Furthermore, as the tenascin meshwork aligns with actin bundles and can modulate the stiffness of the extracellular matrix (Midwood & Schwarzbauer, 2002), actin‐based cell contractility together with modulation of extracellular matrix stiffness has been proposed to play an important role in palatal shelf remodelling and elevation (Chiquet et al., 2016).

Several collagen genes are differentially expressed during palate development and the structural organisation of collagen is important for palatal shelf elevation (Chiquet et al., 2016). Lysyl oxidases (LOXs) modulate tissue rigidity and strength by collagen cross‐linking with *Loxl3* ^−/−^ mice displaying failure of palatal shelf elevation and cleft palate due to changes in collagen structure (Zhang et al., 2015). Similarly, conditional deletion of the Hippo pathway transcriptional co‐activators *Yap*/*Taz* in the palatal mesenchyme resulted in cleft palate with delayed palatal shelf elevation, coincident with reduced expression of *Loxl4* and collagen (Goodwin et al., 2020). As Hippo signalling is mechanosensory, this pathway may be important in mechanical feedback during palatal elevation and extracellular matrix remodelling (Du et al., 2021).

Recent studies have begun to dissect the molecular cross‐talk regulating the extracellular matrix during palate development. Mesenchymal expression of *Tnc* is positively regulated by TGFβ/SHH‐mediated epithelial‐mesenchymal signalling in a stage and domain‐specific manner (Ohki et al., 2020). Furthermore, mice with *Foxf2* deletion displayed reduced expression of extracellular matrix components including *Tnc* and fibronectin, consistent with diminished TGFβ signalling (Nik et al., 2016), while a genome‐wide study of *Foxf2* targets identified several genes involved in extracellular matrix regulation (Xu et al., 2020). Analysis of mice with constitutively active SMO signalling in the palatal mesenchyme also revealed down‐regulation of several medially expressed extracellular matrix proteoglycans concomitant with loss of epithelial *Shh* expression (Hammond et al., 2018). Together these studies indicate that the composition of the extracellular matrix is sensitive to SHH‐SMO signalling, but the underlying mechanisms require further investigation.

Although a role for canonical and non‐canonical WNT signalling has been demonstrated in the palatal epithelium (He et al., 2010a, 2010b, 2011), analysis of mice deficient in components of the non‐canonical WNT/planar cell polarity pathway has revealed an intrinsic role in mesenchymal cell migration and palatal shelf elevation. Mice with loss of *Wnt5a* or its receptor *Ror2* displayed cleft palate with failure of palatal shelf elevation, while compound mutants demonstrated an epistatic effect during palate development (He et al., 2008). Furthermore, *Wnt5a*/*Ror2* regulate chemotactic anterior cell migration of mesenchymal cells in the palate (He et al., 2008). WNT5a can also signal via ROR2/FZD co‐receptor complexes and defects in palatal elevation have been demonstrated in mice deficient for the WNT receptors frizzled 1 (FZD1) and frizzled 2 (FZD2) (Yu et al., 2010). *Fzd2* ^−/−^ mice exhibit cleft palate in ~50% of cases, while *Fzd1* ^−/−^; *Fzd2* ^−/−^ compound mutants display fully penetrant clefts of the secondary palate (Yu et al., 2010). Further evidence for the importance of WNT/planar cell polarity signalling was shown in mice deficient for Prickle1, which displayed cleft palate with palatal shelf elevation defects (Yang et al., 2014). However, further work is needed to determine if Prickle1 mediates the WNT5A/ROR2 signal.

Recently, cranial neural crest‐specific deletion of the WNT receptor *Gpr177*, revealed both canonical and non‐canonical WNT signalling was affected in the anterior mesenchyme during initial palatal outgrowth (Liu et al., 2015). Non‐canonical WNT/planar cell polarity signalling has also been linked to extracellular matrix organisation in the palate. A recent study demonstrated that *Rac1*, an intracellular effector of the PCP pathway, is differentially expressed within the palatal mesenchyme prior to palatal shelf elevation (Tang et al., 2016). Interestingly, *Rac1* overexpression caused shelf elevation defects due to alterations in cell density and fibronectin organisation (Tang et al., 2016). Together, these studies demonstrate the importance of non‐canonical WNT/planar cell polarity signalling in coordinating cell proliferation, migration and physiological changes of the extracellular matrix associated with palatal elevation.

Although the epithelia of the vertical palatal shelves are in direct contact with the mandibular and lingual epithelia, pathological fusion between the palate and the mandible and/or the tongue is rare as the periderm cell layer acts as a protective barrier (Hammond et al., 2019). Periderm forms by initial stratification of the simple ectodermal layer with the onset of desmoglein‐1 expression redistributing membrane tension to promote cellular delamination (Nekrasova et al., 2018). The resulting flattened cells are highly polarised with adhesion complexes excluded from the apical surface of the periderm cells by the ‘fence function’ of tight junctions; as a result, the cells are incapable of adhering to adjacent ectodermal surfaces (Hammond et al., 2019).

Mice carrying mutations in the genes encoding the ligand for the Notch family receptors *Jag2*, the transcription factor *Irf6*, the NF‐κB pathway component *Ikka*, the receptor‐interacting kinase *Ripk4* and the cell cycle regulator *Sfn* display intra‐oral epithelial adhesions that prevent palatal shelf elevation leading to cleft palate (Jiang et al., 1998; Richardson et al., 2009, 2014). In each mutant strain, periderm fails to form and cell adhesion molecules are expressed on the apical surfaces of the exposed basal cells. Similar intra‐oral epithelial adhesions have been observed in *Fgf10*, *Fgfr2*, *Kdf1*, *Grhl3*, *Arhgap29* and *Speccl1* mutant mice (Alappat et al., 2005; Hall et al., 2020; Lee et al., 2013; Paul et al., 2017; Peyrard‐Janvid et al., 2014; Rice et al., 2004).

In humans, failure of periderm formation underlies a series of human congenital disorders that are characterised by multiple inter‐epithelial adhesions and CLP including Van der Woude; popliteal pterygium; Bartsocas Papas; and cocoon syndromes (Hammond et al., 2019). These syndromes arise as the result of mutations in IRF6 (Van der Woude syndrome and popliteal pterygium syndrome); GRHL3 (a subset of IRF6‐negative cases of Van der Woude syndrome); RIPK4 (Bartsocas Papas syndrome); and IKKα (cocoon syndrome; Bartsocas Papas syndrome) (Kalay et al., 2012; Kondo et al., 2002; Lahtela et al., 2010; Leslie et al., 2015; Mitchell et al., 2012; Peyrard‐Janvid et al., 2014).

While the regulatory network controlling periderm formation is incompletely characterised, ΔNp63α, a master regulator of periderm formation, transcriptionally controls *Irf6*, *Ripk4*, *Sfn*, *Grhl3*, *Fgfr2* and *Jag2* (Richardson et al., 2017). In turn, IRF6 promotes periderm differentiation by direct regulation of *Grhl3* and *Klf4* (de la Garza et al., 2013; Liu et al., 2016). However, IRF6 localises to the cytoplasm in an auto‐inhibited state until activated by phosphorylation (De Groote et al., 2015; Kwa et al., 2014). While the kinase function of IKKα is dispensable for keratinocyte differentiation (Hu et al., 2001), RIPK4 phosphorylates IRF6 to drive nuclear translocation and regulate its transactivator activity; mutations in the kinase domain of RIPK4 that underlie Bartsocas Papas syndrome disrupt this activity (De Groote et al., 2015; Kwa et al., 2014). Moreover, RIPK4 is recruited to the LRP6 co‐receptor and phosphorylates DVL proteins after WNT stimulation, leading to stabilisation of β‐catenin and transcription of WNT‐responsive genes (Huang et al., 2013).

## 7 ADHESION AND FUSION OF THE PALATAL SHELVES

**FIGURE 6:**
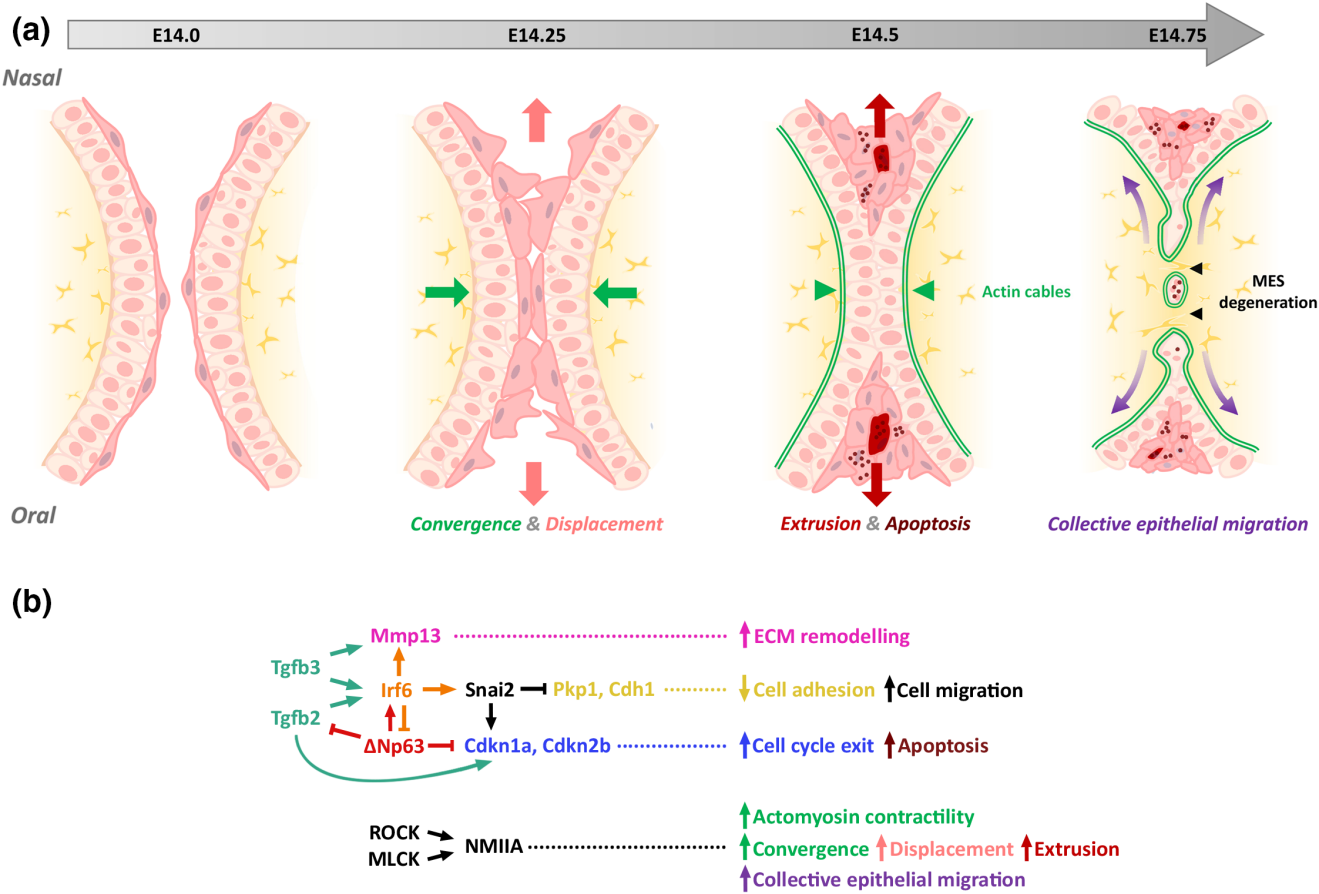
Molecular mechanisms of secondary palate fusion. (a) Schematic diagram illustrating the morphological events in secondary palatal fusion. (b) TGFβ signalling promotes MES degeneration and extracellular matrix remodelling by regulating cascades involving *Irf6* and *Mmp13*, respectively. The transcription factors *Irf6* and *Tp63* function in a regulatory feedback loop to control medial edge epithelial cell fate. Rho‐kinase (ROCK) and myosin light‐chain kinase (MLCK) converge to activate non‐muscle myosin IIA (NMIIA), driving actomyosin contractility. Together this pathway regulates epithelial convergence, displacement, extrusion and migration to ensure midline epithelial seam dissolution and mesenchymal continuity. Adapted from Kim et al., 2015. MEE, medial edge epithelia; MES, midline epithelial seam

After the palatal shelves have elevated, the MEE must rapidly acquire the ability to adhere and fuse if the palatal shelves are not to remain cleft. Competence for palatal shelf adhesion/fusion is precisely regulated; as periderm acts as a barrier which prevents premature adhesion of the oral epithelia, removal of periderm from the MES is a prerequisite for palatal fusion. Initially, periderm was thought to be lost from the surface of the MEE so that initial contact of the palatal shelves was achieved via the exposed basal cells (Fitchett & Hay, 1989; Yoshida et al., 2012). However, other investigators have demonstrated that initial palatal shelf contact and (weak) adhesion is mediated via chondroitin sulphate proteoglycan expressed on the filopodia and lamellipodia that form on the apical surfaces of the periderm cells (Gato et al., 2002; Martínez‐Alvarez et al., 2000; Taya et al., 1999; Vaziri Sani et al., 2010). The periderm cells subsequently migrate out of the MES to form the oral and nasal epithelial triangles (Figure 6) (Cuervo & Covarrubias, 2004; Richardson et al., 2017). Notably, filopodia and lamellipodia are absent, chondroitin sulphate proteoglycan is not expressed, periderm cells fail to migrate from the MES, and the palatal shelves remain cleft in *Tgfb3* ^−/−^ mice (Richardson et al., 2017; Taya et al., 1999).

After periderm removal, the exposed basal cells establish stronger adhesion. Multiple cell adhesion molecules are expressed in the MEE including nectin/afadin family members, cadherins/catenins and tight junctions (Mogass et al., 2000; Richardson et al., 2017; Yoshida et al., 2012); this area has been reviewed comprehensively by Lough and colleagues (Lough et al., 2017). As mice in which the function of these cell adhesion molecules has been genetically modified seldom exhibit cleft palate, the strongest support for their involvement in palatal fusion is provided from human genetics; for example, mutations in *PVRL1* which encodes nectin‐1, *CDH1* which encodes E‐cadherin, and members of the epithelial cadherin‐p120‐catenin complex underlie CLP (Cox et al., 2018; Frebourg et al., 2006; Suzuki et al. 2000). The reasons for the possible differences between mice and humans include early embryonic lethality in mice and differences in the onset of activity in the *Cre*‐driver lines used to generate the genetically modified mice (Lough et al., 2017). To circumvent these problems, an in utero lentiviral‐mediated strategy has been used to analyse the role of the nectin‐afadin cell adhesion complex on palatal development; loss of afadin or combined loss of nectin1/nectin4 function from the palatal epithelia resulted in highly penetrant cleft palate as a result of delayed palatal shelf elevation secondary to intra‐oral epithelial adhesions (Lough et al., 2020). After adhering, the basal cells of the apposed palatal shelves intercalate to form a single‐layered epithelial seam which subsequently degenerates (Figure 6) (Cuervo & Covarrubias, 2004; Kim et al., 2015).

The mechanisms by which MES degeneration occurs have been proposed to include cell migration, apoptosis and epithelial‐mesenchymal transformation; however, the prevailing evidence supports a major role for cell death (Bush & Jiang, 2012). Apoptosis is important in many aspects of organogenesis (Green, 1998). Identification of TUNEL‐ and activated caspase 3‐positive cells in the degenerating MES initially suggested a key role for apoptosis in this process (Bush & Jiang, 2012). Further evidence for this hypothesis is provided by the observations that caspase inhibitors prevent palatal fusion ex vivo (Cuervo et al., 2002); MES degeneration fails to occur in mice that lack the function of the apoptotic regulator APAF1 (Cecconi et al., 1998), and midline defects, including cleft palate, are observed through genetic disruption of apoptosis in *Bok* ^−/−^; *Bax* ^−/−^; *Bak* ^−/−^ mutant mice (Ke et al., 2018). Nevertheless, contradictory results have been reported in that caspase inhibition did not disrupt palate fusion in some studies (Takahara et al., 2004) while palatal fusion has been reported in different *Apaf1* mutant strains (Jin & Ding, 2006).

The issue of whether MES cells undergo epithelial‐mesenchymal transformation remains controversial with some studies supporting this process and others indicating that it does not occur to any great extent (Dudas et al., 2007). Overall, it appears that substantial epithelial‐mesenchymal transformation does not take place with any medial epithelial seam cell migration involving coordinated cell movement along the oronasal axis of the fusing palate to form the epithelial triangles (Carette & Ferguson, 1992; Jin & Ding, 2006; Kim et al., 2015; Logan & Benson, 2020; Richardson et al., 2017). This process of convergent displacement, which is intrinsic to the epithelia, requires the force generated by non‐muscle myosin IIA and upstream regulators of actomyosin contractility (Kim et al., 2015). Recently, apoptosis has been confirmed to be abundant within the MES both before and during its removal; however, completely blocking cell death in the epithelium delays rather than prevents MES removal (Teng et al., 2021). Rather, collective epithelial cell migration occurs with small gaps in the MES consolidating into an interconnected network of epithelial trails that link to the oral and nasal surfaces, and epithelial islands that undergo apoptosis or migrate through the mesenchyme (Figure 6).

As noted above, TGFβ3 plays a key role in MEE fate and palatal fusion. *Tgfb3* expression is activated in future MEE at E13 with abrogation of *Tgfb3* or epithelial‐specific deletion of *Tgfbr1* or *Tgfbr2* resulting in failure of palatal fusion and cleft palate (Dudas et al., 2006; Fitzpatrick et al., 1990; Kaartinen et al., 1995; Pelton et al., 1990; Proetzel et al., 1995; Xu et al., 2006). TGFβ signalling activates both SMAD‐dependent and SMAD‐independent pathways that act partially redundantly to drive MES degeneration (Lane et al., 2015; Xu et al., 2008). TGFβ3‐mediated *Irf6* expression is also crucial for activation of *Mmp13* in, and degeneration of, the MEE; mice carrying loss‐of‐function mutations in *Irf6* failing to undergo MES degeneration and overexpression of *Irf6* in the epithelium rescuing palatal fusion in *Krt14*‐Cre; *Tgfbr2* ^fl/fl^ embryos (Blavier et al., 2001; Iwata et al., 2013; Ke et al., 2015; Knight et al., 2006; Richardson et al., 2009). Moreover, IRF6 and the transcription factor p63 function in a regulatory feedback loop to determine MEE fate: ΔNp63α transcriptionally activates *Irf6* which induces proteasome‐mediated degradation of ΔNp63α (Figure 6) (Moretti et al., 2010; Thomason et al., 2010).

ΔNp63α controls epithelial cell fate to ensure appropriate palatal adhesion (Richardson et al., 2017). Initially, p63 induces the formation and maintenance of periderm (see above). Subsequently, TGFβ3‐induced downregulation of ΔNp63α in the MEE is a prerequisite for palatal fusion by enabling periderm migration to the oral and nasal epithelial triangles and reducing the proliferative potential of the basal layer, in part through regulation of *Cdkn1a* and *Bcl11b* (Iwata et al., 2013; Richardson et al., 2017). Although these processes do not occur in the MEE of *Tgfb3* ^−/−^ mice, downregulation of p63 in *Tgfb3* ^−/−^ embryos restores palatal fusion and ectopic expression of ΔNp63α in wild‐type palatal epithelia causes sub‐mucous cleft palate (Richardson et al., 2017).

While the MES ultimately degenerates, the epithelia on the nasal and oral aspects of the palate differentiate into pseudo‐stratified, ciliated columnar cells and stratified, squamous, keratinising cells, respectively. Although epithelial differentiation is specified by the underlying mesenchyme (Ferguson, 1988), the molecules driving oral and nasal epithelial cell fate are unknown. In addition, the palatal mesenchyme differentiates into bone and muscle to form the hard and soft palate, respectively; the molecular mechanisms specifying the different cell fates have been reviewed recently (Li et al., 2019a, 2019b).

## 8 FUTURE PERSPECTIVES

Although the analysis of genes mutated in syndromic forms of CLP has increased our knowledge of facial development and the molecular pathogenesis of CLP, most cases arise in the absence of additional (non‐cleft) clinical features as non‐syndromic (ns) CLP. While there is a major genetic component to nsCLP (Grosen et al., 2010), the aetiology is complex with multiple interacting genes and environmental factors implicated (Dixon et al., 2011). As a result of this aetiological complexity, genetic approaches to nsCLP have recently focused on genome‐wide association (GWA) studies which have identified ~50 nsCLP susceptibility loci, with several being highly significant in independent studies of diverse populations (Beaty et al., 2010, 2013; Birnbaum et al., 2009; Huang et al., 2019; Leslie et al., 2016, 2017; Ludwig et al., 2012, 2017; Mangold et al., 2010; Sun et al., 2015; Welzenbach et al., 2021). However, while a subset of the associated single nucleotide polymorphisms (SNPs) lie near genes that have been implicated in facial development, the likely causative variant has been identified only at the human chromosome 1q32 locus, where SNP rs642961 disrupts transcription factor AP‐2α binding to an *IRF6* enhancer (Rahimov et al., 2008), and the chromosome 1p22 locus where SNPs rs2275035 and rs4147828 affect the balance of transcription factor binding at regulatory elements controlling *ARHGAP29* expression (Liu et al., 2017).

Genome‐wide association studies indicate that only a small proportion of disease‐associated SNPs result in non‐synonymous changes to protein‐coding sequences suggesting that variants in regulatory sequences contribute markedly to human disease (1000 Genomes Project Consortium, 2010). For nsCLP, the vast majority of GWA signals lie in non‐coding sequence (Ludwig et al., 2017). Methods used to define regulatory elements include identification of sequence conservation (Pennacchio et al., 2006), demarcation of open chromatin conformation (Boyle et al., 2008; Buenrostro et al., 2013), and delineation of histone modifications and bound co‐factors using ChIP‐seq analysis (Heintzman et al., 2007, 2009; Visel et al., 2009). Although initiatives such as the ENCODE, Roadmap Epigenome and GTEx consortia (ENCODE Project Consortium, 2012; GTEx Consortium, 2017; Roadmap Epigenomics Consortium et al., 2015) have collected diverse cells and tissues, characterised the regulatory genome and mapped the effect of genetic variation on molecular traits, the experiments did not capture the entirety of tissue‐specific regulatory activity, particularly that pertaining to facial morphogenesis.

While our current knowledge of facial development and disorders is derived mainly from analysing animal models, differences in the timing of facial development, the underlying molecular architecture and the facial morphology of different species, suggests that animal models are of limited use for interpreting genetic variation in human non‐syndromic CLP. For example, inter‐species differences in gene expression patterns have been demonstrated (Fougerousse et al., 2000); regulatory elements are often poorly conserved even between closely related species with the SNPs associated with human nsCLP frequently absent from their genome (Prescott et al., 2015); and, compared to cleft palate, cleft lip is rarely observed in model organisms.

To overcome these limitations, transcriptomic and epigenomic analyses of human cells and bulk tissues have been undertaken using early human neural crest cells (Rada‐Iglesias et al., 2012); lineage‐specified human cranial neural crest cells (Prescott et al., 2015) and embryonic mid‐facial tissue obtained from Carnegie Stage (CS)13‐CS17 embryos (Wilderman et al., 2018). These studies demonstrated a significant enrichment of nsCLP GWAS‐associated variants in active chromatin regions from both human neural crest cells and mid‐facial tissue (Ludwig et al., 2017; Welzenbach et al., 2021; Wilderman et al., 2018) and allowed the prediction of candidate genes for developmental syndromes that include cleft palate (Gerrard et al., 2016, 2020). However, as outlined above, facial processes and palatal shelves exhibit spatio‐temporal heterogeneity of gene and regulatory element expression; as a result, these studies merged the gene expression and chromatin states of different cell types in the developing face. Future studies to address these issues using single‐cell RNA‐seq (transcriptomic), single‐cell ATAC‐seq (epigenomic) and multi‐omic approaches are essential to define the cell‐type‐specific gene regulatory networks driving normal embryonic facial development and how they are disrupted in both syndromic and non‐syndromic CLP.

## CONFLICT OF INTEREST

The authors have declared that no conflict of interest exists.

## AUTHOR CONTRIBUTIONS

**Nigel Lowe Hammond:** Writing – original draft; Writing – review & editing. **Michael Dixon:** Writing – original draft; Writing – review & editing.

### PEER REVIEW

The peer review history for this article is available at https://publons.com/publon/10.1111/odi.14174

## Data Availability

Data sharing is not applicable to this article as no new data were created or analyzed in this study.
